# Trends in inpatient orthopedic surgery during the COVID-19 pandemic in Japan: a nationwide data study

**DOI:** 10.1186/s12891-024-07620-w

**Published:** 2024-06-27

**Authors:** Mitsuhiro Nishizawa, Kosei Nagata, Babapelumi Adejuyigbe, Tomohiro Shinozaki, Koji Yamada

**Affiliations:** 1 Orthopaedic Trauma Institute, University of California, San Francisco, CA USA; 2 Department of Orthopaedic Surgery and Spinal Surgery, The University of Tokyo, 7-3-1, Hongo, Bunkyo-Ku, Tokyo, 113-8655 Japan; 3 David Geffen School of Medicineat , The University of California, Los Angeles, CA USA; 4 Department of Information and Computer Technology, Faculty of Engineering, Tokyo University of Science, Tokyo, Japan; 5 Nakanoshima Orthopaedics, 1F, F&F Haimu, 6-26-2, Nakanoshima, Tama-ku, Kawasaki, Kanagawa Japan

**Keywords:** Orthopedic surgery, COVID-19, Statement of Emergency, National Database of Health Insurance Claims and Specific Health Checkups of Japan

## Abstract

**Background:**

Coronavirus disease 2019 (COVID-19) has resulted in substantial morbidity and mortality globally. The National Database of Health Insurance Claims and Specific Health Checkups of Japan (NDB) covers 99.9% of health insurance claim receipts by general practitioners. The purpose of this study is to investigate the nationwide number of inpatient orthopedic surgeries in Japan during the effect of state of emergency (SoE) due to COVID-19.

**Method:**

The NDB has been publicly available since 2014. We retrospectively reviewed the NDB from April 2019 to March 2022. We gathered the monthly number of all inpatient orthopedic surgeries. We also classified orthopedic surgeries into the following 11 categories by using K-codes, Japanese original surgery classification: fracture, arthroplasty, spine, arthroscopy, hardware removal, hand, infection/amputation, ligament/tendon, tumor, joint, and others. By using the average number from April to December 2019 as the reference period, we investigated the increase or decrease orthopedic surgeries during the pandemic period.

**Results:**

The NDB showed that the average number of total inpatient orthopedic surgeries during the reference period was 115,343 per month. In May 2020, monthly inpatient orthopedic surgeries decreased by 29.6% to 81,169 surgeries, accounting for 70.3% of the reference period. The second SoE in 2021 saw no change, while the third and fourth SoEs showed slight decreases compared to the reference period. Hardware removal and tumor surgeries in May 2020 decreased to 45.3% and 45.5%, respectively, while fracture surgeries had relatively small decreases.

**Conclusion:**

According to NDB, approximately 1.3 million orthopedic inpatient surgeries were performed or claimed in a year in Japan. In May 2020, the first SoE period of the COVID-19 pandemic, the number of inpatient orthopedic surgeries in Japan decreased by 30%. Meanwhile, the decrease was relatively small during the SoE periods in 2021.

## Introduction

The burden of The Coronavirus Disease (COVID-19) Pandemic has left an indelible impact on populations and public health systems across the globe, profoundly impacting morbidity and mortality worldwide [1]. In December 2019, beginning with the reporting of several patients with severe pneumonia of ‘unknown etiology’ in Wuhan, China – the characterization of the disease was quickly re-classified to a pandemic by the World Health Organization (WHO) in a matter of months [2]. On 11 February 2020, the WHO announced that the disease caused by the novel coronavirus would be named COVID-19 [1]. One month later, on 11 March 2020, the WHO declared that COVID-19 should be characterized as a pandemic.

Both emergent and non-emergent surgery became a major risk factor due to the possibility of infection and postoperative pulmonary complications for patients with and SARS-CoV-2 [3]. The Japanese Orthopaedic Association (JOA) declared that orthopedic surgeries should be postponed during the pandemic except for conditions requiring early intervention, such as paralysis, trauma, open fractures, and malignancy [4]. Such conditions for surgery were primarily upheld during periods deemed to be a ‘State of Emergency (SoE) by the Japanese government. In Japan, four SoEs were declared at different times in the pandemic, between 2020–2021. Though the specifics of these SoEs varied between prefectures, these periods were from (1) 4/7/2020 – 5/25/2020; (2) 1/8/2021 – 3/21/2021; (3) 4/25/2021 – 6/20/2021; and (4) 7/12/2021 – 9/30/2021 universally. Though the recent report showed that there was statistical decrease in the number orthopedic surgery during pandemic [5], the nationwide effects in Japan has not been well discussed.

For more than 60 years, the Japanese healthcare system is characterized by universal insurance coverage [6]. These characteristics enable us to estimate the total number of specific surgeries by analyzing Japanese inpatient claims data [7, 8]. Since 2014, the Ministry of Health, Labour and Welfare (MHLW) has released data from the National Database of Health Insurance Claims and Specific Health Checkups of Japan (NDB) [9]. Using inpatient claims data from this database, it is possible to monitor and estimate the total number of specific completed at various time periods in Japan on a national scale. Although it does not provide data on the severity of individual patients’ conditions or patients’ clinical outcomes [10], the NDB has covered 99.9% of public health insurance claims from hospitals and 97.9% from clinics [11]. Using the NDB, this study aims to evaluate the impact of the COVID-19 pandemic on the epidemiology of orthopedic surgeries during the SoE periods and illuminate the intricate interplay between the COVID-19 pandemic and the patterns of orthopedic surgeries in Japan.

## Methods

### NDB files

Since 2014, the MHLW has monitored and released data on the number of different types of surgeries completed in Japan each fiscal year [9]. Each Japanese fiscal year begins April 1 and ends March 31, the following year. In this study, inpatient data of the fiscal years of 2019, 2020, and 2021 were used for analysis. including monthly surgical claims data from April 2019 to March 2022, as monthly data was available starting in 2019. The data is compiled into an Excel© file for each fiscal year—which is available for download. The specifics of the data include the number, as well as the types of different surgeries completed for each fiscal year, if there were fewer than 10 cases in one category, the data was not shown. This missing data does not introduce a significant bias to the overall results, as the large number of cases categorized as "Others".

### Period of state of emergency

A SoE can be defined as a legal declaration by a government indicating that it faces a situation of crisis or disaster and, as a result, grants specific powers and authority to relevant officials or agencies to respond effectively to the emergency. During a state of emergency, governments often can implement extraordinary measures, such as restrictions on movement, curfews, and allocation of resources to address the crisis. This declaration is typically temporary and allows for swift, coordinated, and sometimes extraordinary actions to safeguard public safety and well-being. We used information from the Cabinet Secretariat to determine periods of SoE in Japan during the pandemic [12]. For this study, these periods were determined to be from: (1) 4/7/2020 – 5/25/2020; (2) 1/8/2021 – 3/21/2021; (3) 4/25/2021 – 6/20/2021; and (4) 7/12/2021 – 9/30/2021.

### Monthly trend of the number of each surgery using K-codes

We investigated the number of orthopedic surgeries based on K-code data in NDB files. A K-code is a unique procedure classification used by the MHLW in Japan [7, 8, 10, 13]. Raw data is presented in Supplementary Table 1. Briefly, orthopedic surgeries are classified from K023 to K114 in this NDB system. First, we counted the total number of orthopedic surgeries per month to evaluate the monthly trend. The number of surgeries was graphed including information on the period when the SoE was declared. Next, following K-code information, the orthopedic surgeries were classified into the following 11 categories by using K-codes: fracture, arthroplasty, spine, arthroscopy, hardware removal, hand, infection/amputation, ligament/tendon, tumor, joint, and others. We also graphed each category data with the SoE period as well.

We calculated the average monthly number of surgeries for each category from April to December 2019 as the reference period, to further evaluate the effect of the SoE declaration, the percentage increase or decrease of each category in each month was calculated from January 2020 to March 2022 in comparison to the pre-pandemic reference data. The number of surgeries during each SoE period was estimated by dividing the monthly surgeries by the number of days in the SoE period. Subsequently, the average number of surgeries per day within each SoE were calculated For the analysis of tumor surgeries, we analyzed malignant tumors separately with K031, K053, K136 codes and benign tumors K030, K052, K135 codes.

### Statistical analysis

Given the comprehensive nature of the survey, no inferential statistical measures such as p-values or confidence intervals were calculated.

## Results

**Fig. 1:**
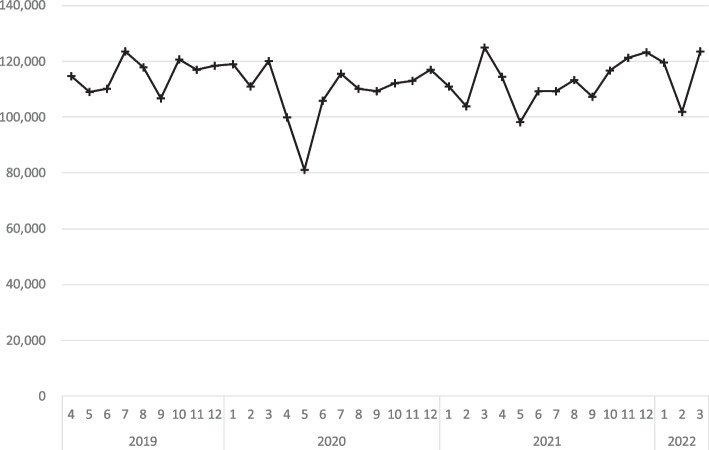
The trends in the number of monthly inpatient orthopedic surgeries. Vertical axis indicates number of cases per month. Horizontal axis means year and month, and gray bar means the duration of the declared state of emergency in Japan

The trends in the number of monthly inpatient orthopedic surgeries can be seen in Fig. 1. Periods of SoE have been represented as gray bars. Each point is located in the middle of the subsequent month. Before the period of SoEs began, there were between 106,794 and 123,383 inpatient orthopedic surgeries in the reference period from April to December 2019. The average number of total inpatient orthopedic surgeries was 115,343 per month in reference period. The annual number of surgeries in the three years before 2019 was 1,263,963 in 2016, 1,331,223 in 2017, and 1,367,871 in 2018. The monthly averages were 105,330 in 2016, 110,935 in 2017, and 113,989 in 2018, respectively; these figures were within 10% of the reference figures. In May 2020, the month with the lowest number of surgeries in the first SoE (4/7/2020–5/25/2020), the number of monthly inpatient orthopedic surgeries decreased by 29.6% to 81,169—amounting to 70.3% of the number of orthopedic surgeries observed during reference. In June 2020, following the first SoE, the number of inpatient orthopedic surgeries rose to105,939. During the second period of SoE (1/8/2021–3/21/2021) the month with the lowest number of surgeries was February with 103,944 inpatient orthopedic surgeries, amounting to 90.1% of the reference period. In the third SoE (4/25/2021 – 6/20/2021), the number of inpatient orthopedic surgeries was the lowest in May, with 98,056 – amounting to 85% of the reference period. Lastly, during the final period of SoE (7/12/2021 – 9/30/2021) the lowest number of inpatient orthopedic surgeries was 107,225 in September – amounting to 93.0% of reference period.

The SoE period covered a total of 259 days, during which it is estimated that a total of 904,859 surgeries were performed. The average number of surgeries per day was 3,494, which amounted to 92.6% of the reference period. The estimated number of surgeries for each SoE period was as follows: 145,470 (49 days) for the first period, 274,515 (72 days) for the second period, 193,791 (57 days) for the third period, and 291,084 (81 days) for the fourth period. The average number of surgeries per day during each SoE period was as follows: 2,969 (78.6%) in the first period, 3,813 (101.1%) in the second period, 3,400 (90.1%) in the third period, and 3,594 (95.2%) in the fourth period, compared to the reference period (Table 1),

**Table 1:** Average number of surgeries per day in Japan

	Days	Estimated number of surgeries per day	%
Reference period			
04/2019 – 12/2019	275	3,775	Ref
SoE total	259	3,494	92.6
The first SoE			
04/07/2020 – 05/25/2020	49 (actual)	2,969	78.6
From April to May 2020	61 (using data set)		
The second SoE			
01/08/2021 –03/21/2021	72 (actual)	3,813	101.0
From January to March 2021	90 (using data set)		
The third SoE			
04/25/2021 – 06/20/2021	57 (actual)	3,400	90.1
From April to June 2021	90 (using data set)		
The fourth SoE			
07/12/2021 – 09/30/2021	81 (actual)	3,594	95.2
From July to September 2021	92 (using data set)		

*SoE* State of emergency

**Fig. 2:**
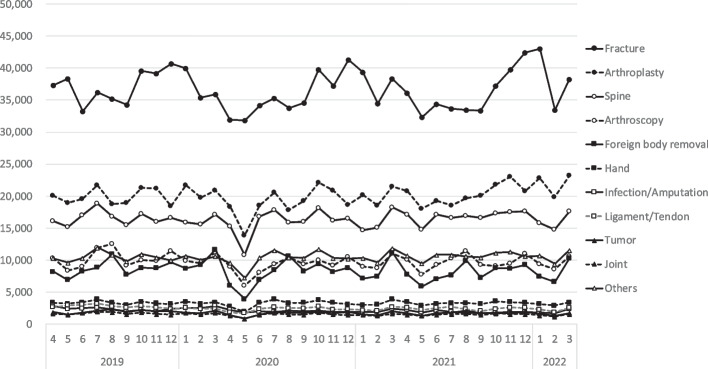
The monthly number of surgeries for by orthopedic surgical category. The vertical axis shows the monthly number of cases in each category. Horizontal axis means year and month, and gray bar means the duration of the declared state of emergency in Japan

**Fig. 3:**
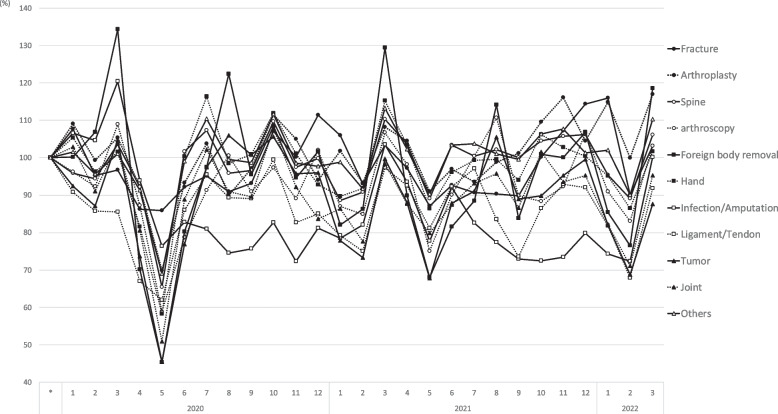
The respective increases/decreases in the number of different orthopedic surgeries. The vertical axis shows the increase or decrease in the number of surgeries in each category from the reference period, expressed as a percentage. Horizontal axis means year and month, and gray bar means the duration of the declared state of emergency in Japan

**Fig. 4:**
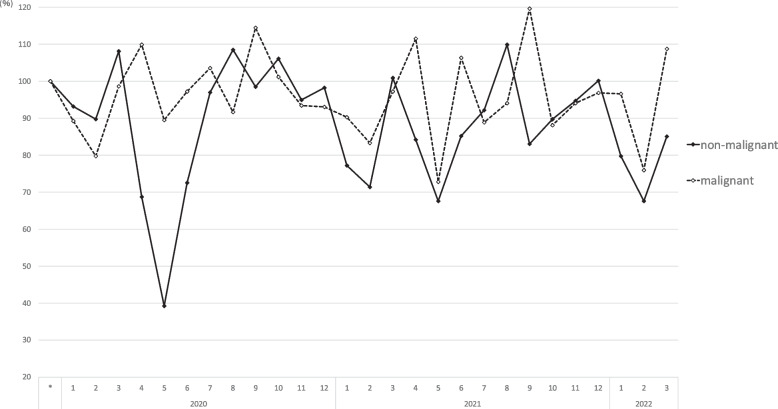
The respective increases/decreases in the number of orthopedic surgeries for benign and malignant tumor. The vertical axis shows the increase or decrease in the number of benign and malignant tumor surgeries from the reference period, expressed as a percentage. Horizontal axis means year and month, and gray bar means the duration of the declared state of emergency in Japan

Figure 2 shows the monthly number of surgeries for by orthopedic surgical category. The respective increases/decreases in the number of different orthopedic surgeries are represented in Fig. 3 in comparison to the reference period. Additionally, when compared with the reference period, the number of (complex/routine) surgeries eg. hardware removal surgeries and tumor resection surgeries decreased to 45.3% and 45.5%, respectively, in May 2020 (Fig. 3). Considering the classification of "tumors" into benign and malignant, the number of surgeries in May 2020 was 39.3% for benign tumors and 89.6% for malignant tumors compared to the reference period (Fig. 4). The decline in fracture and infection/amputation surgeries was relatively small, to 86.0% and 76.4%, respectively.

Observations by fiscal year showed that there were 1,388,354, 1,315,498, and 1,357,383 inpatient orthopedic surgeries in the 2020, 2021, and 2022 period (March–April), respectively. The actual year-by-year observations showed that there were 1,325,869 and 1,352,526 surgeries in 2020 and 2021, respectively.

## Discussion

This study used NDB and showed the annual number of inpatient orthopedic surgeries performed in Japan on a national scale. Approximately 1.3 million orthopedic inpatient surgeries were performed or claimed in a year in Japan. The universal health insurance coverage in Japan enables us to gather nationwide comprehensive data from the perspective of national welfare [6]. Considering that there is a 99.9% coverage of claims within the NDBs database, it is reasonable to use the NDB to obtain nationwide data on the total number of orthopedic surgeries in Japan [11]. We investigated the number of inpatient orthopedic surgeries from April 2019 to March 2022. In the fiscal year 2020, the number of surgeries in Japan seemed to be influenced by the COVID-19 pandemic. There was an approximate 30% reduction in the number of inpatient orthopedic surgeries in May 2020.

Okamoto et al. reported that the number of surgeries in Japan was influenced by the COVID-19 pandemic in the fiscal year 2020 [14]. This reduction in case volume led to decreased surgical experience for trainees [15, 16]. In the acute phase of COVID-19 pandemic, the number of orthopedic operations performed decreased by 60% in Poland [16]. In the U.S., the volume of elective spine surgeries decreased by 21.6% in 2020 after the pandemic, compared with the calendar year 2019 [17]. These countries recover the number of surgeries after 2021.

In response to the SoE, the JOA declared that all but the most urgent surgeries should be postponed during pandemic [4]. In May 2020 in the first SoE period of the COVID-19 pandemic, the largest decrease was observed, with the number of surgeries decreasing 30% compared to the number of referrals. This decrease in the number of surgeries aligns with the JOA policy. Conversely, the reduction in the number of surgeries during the third and fourth SoE periods was relatively modest, with a decrease of 5–10%. In the second SoE period, there was no decrease—and the number of surgeries increased to a frequency even slightly higher than reference period, registering at 101%. The duration of the SoE periods also appear to have influenced the average number of surgeries per day, where a shorter duration exhibited a more pronounced impact on orthopedic healthcare. In contrast, periods lasting longer than two months do not seem to have had a significant impact on the number of orthopedic surgeries performed. This trend may be attributed to familiarity with declaring the SoE over time, or to heightened concerns about hospital operations.

This study showed that the extent of decrease in the number of surgeries for trauma or infections/amputations was relatively small. Considering Topcu's findings, which reported a 17.63% decrease in total surgical procedures but a 71.43% increase in emergency surgeries during the pandemic, our results may reflect the urgency of these surgeries [18]. The decrease in the number of arthroplasty surgeries could vary with a more detailed classification of surgical procedures. Given that the pandemic did not alter the epidemiology of hip fractures, excluding bipolar hip arthroplasty—which is more common among older trauma patients and performed without delay—from this category might have revealed a more pronounced decrease in elective arthroplasty surgeries [19, 20]. However, the original data in this study did not allow for distinguishing whether bipolar hip arthroplasty was performed for femoral neck fractures or elective arthritis surgery.

This study used NDB and showed the annual number of inpatient orthopedic surgeries performed in Japan on a national scale. Approximately 1.3 million orthopedic inpatient surgeries were performed or claimed in a year in Japan. The number of orthopedic surgeries in the U.S. was assumed to be 7–18 million a year in the United States [21, 22]. It is difficult to gather nationwide number of surgeries in the U.S. because it is a patchwork of private insurance companies and public programs to get a handle on how much is spent [21]. Even in Germany, it is also difficult to investigate reliable data on the total number of orthopedic surgeries at the national level due to the mixture of public and private insurance coverage [23]. The unique system of Japanese insurance system enabled this study, which we believe is the first report covering various procedures nationwide using one of the most reliable real-world data, regarding the impact on the number of surgeries during the COVID-19 pandemic. We considered that the number of inpatient orthopedic surgeries in Japan, 1.3 million per year, was reliable because of Japan’s universal insurance system [6] and the high NDB database cover rate, as 99.9% [11].

There are several limitations in this report. First, this NDB Excel© sheet presented monthly data. As the periods of SoE spanned the course of several months – sometimes beginning in the middle of the month and ending in the middle of the month, the unavailability of daily data creates slight inaccuracies in our analysis. Additionally, more granular, detailed, categorical data for the (types of different surgeries completed?) were not available from the NDB. Moreover, categories with less than 10 surgical cases were excluded from our counts. Therefore, these uncounted variables decrease accuracy slightly as well. In addition, the reference period of this study is controversial. We set the average number from April to December 2019 as the reference, because COVID-19 first made headlines in Japan with the arrival of the Diamond princess in February 2020 [24]. Although the number of surgeries during the reference period in this study is not significantly different from the years before the pandemic, the shape of the graph in Fig. 3 may vary depending on the chosen reference period. Moreover, due to the fact that we focused primarily the epidemiological data, no correction was made for the number of days. For example, February has 29 days in 2020 and 28 days in 2021 and 2022. Additionally, there are also many holidays in May in Japan – which would have confounding effects on productivity during this period. Despite these limitations, we expect that this report using the NDB presented epidemiological data. Despite its limitations, this cross-sectional study is the first in Japan to investigate the impact of declaring a state of emergency due to a global viral pandemic. Findings from this study provide insight into the impact of such a crisis and will be valuable for future preparedness.

## Conclusion

The NDB data showed that approximately 1.3 million orthopedic inpatient surgeries were performed in a year in Japan during the COVID-19 pandemic. In May 2020 in the first SoE period of the COVID-19 pandemic, the number of inpatient orthopedic surgeries in Japan decreased by approximately 30%. Large decrease was observed especially in hardware removal and tumor surgeries. The decrease in fracture surgeries was relatively small. Meanwhile, the decreases of the number of orthopedic surgeries were relatively small during the second, third, and fourth SoE periods in 2021.

## Data Availability

The datasets generated and/or analyzed during the current study are publicly available. Original NDB data is open in https://www.mhlw.go.jp/stf/seisakunitsuite/bunya/0000177182.html.

## Abbreviations

COVID-19: Coronavirus disease 2019
JOA: Japanese Orthopaedic Association
MHLW: Ministry of Health, Labour and Welfare
NDB: National Database of Health Insurance Claims and Specific Health Checkups of Japan
SoE: State of emergency
WHO: World Health Organization
